# CARD9 deficiency predisposing chromoblastomycosis: A case report and comparative transcriptome study

**DOI:** 10.3389/fimmu.2022.984093

**Published:** 2022-09-09

**Authors:** Chen Huang, Weiwei Deng, Yi Zhang, Kai Zhang, Yubo Ma, Yinggai Song, Zhe Wan, Xiaowen Wang, Ruoyu Li

**Affiliations:** ^1^ Department of Dermatology and Venerology, Peking University First Hospital, Beijing, China; ^2^ Research Center for Medical Mycology, Peking University, Beijing, China; ^3^ Beijing Key Laboratory of Molecular Diagnosis on Dermatoses, Beijing, China; ^4^ National Clinical Research Center for Skin and Immune Diseases, Beijing, China

**Keywords:** chromoblastomycosis, dematiaceous fungi, immunity, CARD9, *Phialophora expanda*

## Abstract

*CARD9* mutations are known to predispose patients to phaeohyphomycosis caused by different dematiaceous fungal species. In this study, we report for the first time a patient of chromoblastomycosis caused by *Phialophora expanda*, who harbored *CARD9* mutation. Through a series of *in vivo* and *in vitro* studies, especially a comparative transcriptome study, we compared this case with our former patient suffering from phaeohyphomycosis caused by *Phialophora americana*. We showed that *P. expanda* is prone to forming sclerotic bodies both *in vitro* and in *Card9* knockout mice, and has a stronger immunogenicity than *P. americana*. These data preliminary demonstrated that besides host defense, fungal specificity also contributed to the clinical phenotype in *CARD9* deficient patients with dematiaceous fungal infections.

## Introduction

Dematiaceous fungi, also known as phaeohyphomycetes or melanized fungi, are so named owing to the dark pigmentation in the walls of their hyphae and/or spores (1, 2). They are associated with various clinical manifestations, mainly chromoblastomycosis and phaeohyphomycosis. Histologically, chromoblastomycosis is characterized by the presence of sclerotic or muriform cells, while phaeohyphomycosis is characterized by yeast-like, pseudo-filamentous, or filamentous components in tissue.

Since our first report on subcutaneous phaeohyphomycosis in CARD9 deficient patients in 2014, many studies have reported the link of CARD9 deficiency to phaeohyphomycosis, in both subcutaneous and invasive infections (3). Moreover, our previous study in *Card9* knock-out mice further confirmed the susceptibility of Card9 deficient mice to phaeohyphomycosis caused by many dematiaceous fungal species (4). It is considered that CARD9 deficiencies are prone to causing phaeohyphomycosis in dematiaceous fungal infections, since the defective host immune responses favor fungal growth in the mycelium form in tissue instead of sclerotic bodies. We used to hypothesize that chromoblastomycosis patients are relatively immunocompetent since it has never been reported in patients with genetic defects.

In this study we report, for the first time, a patient harboring the *CARD9* mutation with chromoblastomycosis caused by *Phialophora expanda*, which is a fungus from the *Phialophora verrucosa* complex according to our previous phylogenetic studies (5). We designed a series of *in vivo* and *in vitro* studies to compare this case with our former patient suffering from phaeohyphomycosis caused by *P. americana*, from the same complex (5). We preliminarily demonstrated that besides host defense, fungal specificity also contributed to the clinical phenotype in patients with dematiaceous fungal infections.

## Materials and methods

### Ethics

Two patients in this study, their family members, and six ethnically matched healthy volunteers provided written informed consent for participation in the study, which was approved by the Clinical Research Ethics Committee of the Peking University First Hospital. The patients permitted us to use their images and medical information.

### Pathogen DNA extraction, amplification, and sequencing

By use of the DNeasy Plant Mini Kit (Qiagen, Hilden, Germany), genomic DNA was extracted and purified from approximately 1 cm^2^ of fungal elements. Cells were disrupted with glass beads (425–600 µm) (Sigma-Aldrich, Zwijndrecht, The Netherlands) and TissueLyser II (Qiagen). The nuclear genes, ITS and BT2, were amplified by PCR. Details of PCR amplification and sequencing primers can be found in the reference paper (6). Amplification was performed with the 2×EasyTaq PCR SuperMix protocol (Trans Gen Biotech, Beijing, China). Add template DNA (50–100 ng) and forward and reverse primers (0.2–0.4 µM each) to a total reaction volume of 25 µL. DNA amplification was performed in a Mastercycler (Eppendorf, Hamburg, Germany), the process of which includes machine-preheating at 94°C for 5 min, 30 cycles of denaturation at 94°C for 30s, annealing at 54°C for 30s, extension at 72°C for 30s, and final extension at 72°C for 10 min. Amplified bands were visualized using the Gel Doc XR+ system (BioRad, Hercules, CA, USA) with Trans2K Plus DNA Marker (Trans Gen Biotech) indicating size and concentration. PCR products were sequenced by Sangon Biotech Co. Ltd. (Shanghai, China). The alignments and phylogenetic reconstructions were performed in accordance with Li et al. (5).

### Isolation of human peripheral blood mononuclear cells

Human peripheral blood mononuclear cells (PBMCs) were collected from whole blood by density-gradient centrifugation using Ficoll-Paque Plus (GE Healthcare, Chicago, IL, USA) as previously described (6). Specifically, fresh venous blood was drawn into 10 mL EDTA tubes and diluted with phosphate-buffered saline (PBS). PBMCs were isolated using Ficoll-Paque density-gradient centrifugation, meanwhile, erythrocytes in the pellet were lysed. PBMCs were flushed twice with PBS at 800 × g for 8 min and reconstituted in RPMI 1640 medium (HyClone, Logan, UT, USA) supplemented with 100 U/mL penicillin and 100 μg/mL streptomycin (HyClone).

### Peripheral blood mononuclear cells stimulation assays

*P. expanda* isolates were cultured on potato dextrose agar (PDA; BD Biosciences, San Jose, CA, USA) for 3–14 days at 28°C to harvest conidia. Heat-killed (HK) conidia for stimulation were prepared for 30 min at 99°C in a water bath.

The PBMCs were incubated in 96-well plates at a final concentration of 2.5 × 10^6^/mL in a total volume of 200 μL RPMI 1640 medium with 10% autologous serum per well. The PBMCs were stimulated with different pattern recognition receptor agonists, including lipopolysaccharide (LPS, a Toll-like receptor 4 agonist, 100 ng/mL), Trehalose-6,6-dibehenate (TDB, a Mincle agonist, 100 μg/mL), β-glucan (a Dectin-1 agonist, 50 μg/mL), and fungal particles (HK or viable resting conidia, 10^7^ particles/mL) at 37°C in a 5% CO_2_ atmosphere. Innate ELISA assays were carried out with stimulated culture supernatants or cells after 24 h. In order to detect the adaptive immune response, PBMCs were stimulated with the corresponding HK fungal particles for 6 days, and cells or culture supernatants were gathered for further ELISA assays and FACS.

### Cytokine measurements and intracellular cytokine staining of CD4^+^ T cells

After human PBMCs were stimulated for 24 h or 6 days, cell culture supernatants were collected and stored at -70°C. According to the instructions provided by the manufacturer, we used commercial ELISA kits (R&D Systems, Minneapolis, MN, USA) to measuring the cytokines of the patients and healthy controls.

PBMCs were incubated with different HK fungal stimuli for six days, then residual living cells were re-stimulated with Cell Stimulation Cocktail (eBioscience, San Diego, CA, USA) for 5 h in an incubator to promote the intracellular accumulation of secreted cytokines. Human PBMCs were surface stained with FITC-conjugated anti-human CD4 antibody, fixed and permeabilized by Cytofix/Cytoperm solution (BD Biosciences), and stained with PE-conjugated anti-human IL-22 and Alexa^®^Fluor647-conjugated anti-human IL-17A antibodies. Data were collected on a BD FACS Calibur system and analyzed with FlowJo7.6 software.

### RNA-sequencing (RNA-seq) analyses and immunohistochemical analysis

To explore the differential transcript profile between the two patients, we conducted RNA-seq using the Illumina platform. We used the biopsy specimens from skin lesions of the patients to extract RNA using Total RNA Kit I (R6834-01, OMEGA, USA). Raw counts were normalized to balance the sequencing depth using fermented palm kernel meal (FPKM). Then, the parameters (q ≤0.01 and |log_2_ Fold change| ≥1) were used to identify differentially expressed genes (DEGs, Patient2 versus Patient1) using the Limma package (version 3.42.2) in R. To reveal the pathway activities of DEGs, we performed pathway enrichment analyses for up-regulated DEGs (q ≤0.01 and log_2_ Fold change ≥1) and down-regulated DEGs (q ≤0.01 and log_2_ Fold change ≤-1) using the ClusterProfiler package (version 3.14.3). Antifungal immunity-related genes were obtained from previous studies. Default statistical methods in respective packages or software were used to conduct the test the significance.

The biopsy specimens from skin lesions of the two patients were fixed in 10% buffered formalin and paraffin embedded for immunohistochemical analysis. According to the instructions of the IHC antibody, we performed immunohistochemical staining. Stained slides were photographed by microscope.

### Murine model of subcutaneous dematiaceous fungal infection

*Card9*-KO mice (C57BL/6 background) were generously provided by Xin Lin (Tsinghua University School of Medicine, Beijing, China, and MD Anderson Cancer Center, Houston, TX). In this study, 6−8-week-old *Card9*-KO and C57BL/6 WT mice (Vital River Laboratories, Beijing, China) were maintained in specific pathogen-free facilities at the Institute of Clinical Pharmacology of Peking University. The WT and *Card9*-KO mice were injected at two hind footpads subcutaneously with 100 μL viable *P. expanda* (1×10^9^ particles/mL). Skin biopsy specimens were obtained, and slides were stained with periodic acid-Schiff for histopathological analysis.

### *In vitro* induction of muriform cells

Main components of *in vitro* culture medium were MgSO_4_·7H_2_O, KH_2_PO_4_, NH_4_NO_3_, Biotin, Thiamine, Glycerol, and Nicomycin; pH:5.5−6.0. To this medium, 100 μL viable *P. expanda* spores (1×10^7^/mL) were added and placed in an incubator for 45−50 days, setting the temperature to 35−36 °C.

### Statistical analysis

Data were plotted using GraphPad Prism 7.0 software (La Jolla, CA, USA) and analyzed with unpaired *t*-tests in SPSS 22.0 software (Chicago, IL, USA). *P* values of <.05 were considered statistically significant.

## Results

### Similar clinical findings in two CARD9-deficient patients with chromoblastomycosis and phaeohyphomycosis

**Figure 1:**
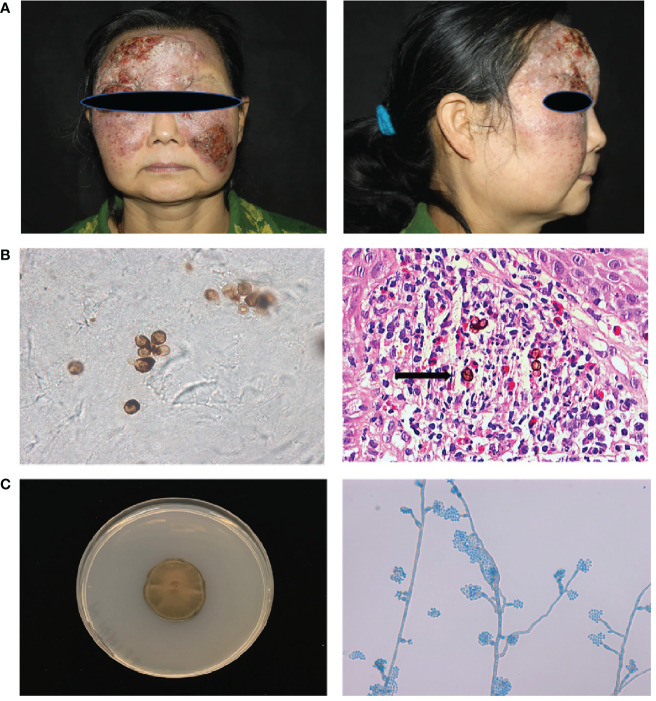
Clinical features, laboratory findings of patient 1. **(A)** A protuberant dark red mass on the face with erosion, crust, and pus, which covered the forehead and cheek with a clear boundary. **(B)** Smear of the lesion scrapings showed brown sclerotic cells with cross septa. Histopathological examination of the skin lesion showed pseudoepitheliomatous hyperplasia of the epidermis, and intense dermal inflammatory infiltrations with the presence of sclerotic cells in the dermis (Haematoxylin and eosin stain; 400× original magnification) **(C)** Macroscopic appearance of the cultured pathogen: the colony was brown to blackish‐green with abundant short, grey aerial hyphae (Potato dextrose agar, 14 d at 28°C). Microscopic examination of the cultured organisms revealed vase-shaped sporogenous cells with collar-like structure and flower-like arrangement of small conidia.

Patient1(P1) was a 55-year-old woman from northeast China, born to non-consanguineous parents. She presented to our clinic with an erythematous plaque on her face, that had been gradually enlarging for the past 30 years. At the age of 23, she noted several red papules on her forehead; the area gradually expanded with occasionally itching and without pain. She was diagnosed with “fungal granuloma” at a local hospital and treated with “itraconazole 400 mg/day” for approximately 1.5 years. However, the skin lesions enlarged and spread to her forehead, cheek, and eyelid. Dermatological examination revealed a protuberant dark red mass on her face with erosion, crust, and pus, which covered the forehead and cheek with a clear boundary (**Figure 1A**). A skin biopsy from the lesion showed pseudoepitheliomatous hyperplasia of the epidermis and intense dermal inflammatory infiltrations with the presence of multiple sclerotic cells within the dermis. (**Figure 1B**) A smear of the lesion scrapings showed brown sclerotic cells with cross septa (**Figure 1B**). Therefore, a diagnosis of chromoblastomycosis was made.

Cultures of the biopsied tissue specimens showed filamentous fungus, whose colony was dense, brown to black after 21 days on potato dextrose agar at 28 °C. Further microscopic examination of the cultured organisms revealed vase-shaped sporogenous cells with collar-like structures and flower-like arrangements of small conidia, which implied it belongs to *Phialophora* spp. (**Figure 1C**). According to our previous phylogenetic analysis of *Phialophora verrucosa* complex, there are seven species in this complex, including *P. verrucosa, P. americana, P. expanda, P. chinensis, P. tarda, P. ellipsoidea and P. macrospora* (5). To further identify the isolate, we sequenced the *ITS* and *BT2* rRNA genes using PCR. Then, we constructed the phylogenetic tree based on *ITS* and *BT2* and found this species belonged to the *P. expanda* clade, which has never been reported to cause disease in the literature. Amphotericin B plus itraconazole was used for therapy. There were some improvements after treatment, which to some extent control the progress of the disease, but she was not completely cured and was still under treatment. Based on previous reports and the recalcitrant character of this patient, we extracted her peripheral blood DNA to sequence for *CARD9*‐coding exons, and found a homozygous frameshift mutation in exon 6 (c.819‐820insG, p. D274fsX60).

Patient2 (P2) was a male with previously reported phaeohyphomycosis caused by *P. americana*, also from the *P. verrucosa* complex (5, 6). Besides his similar facial rash, his *CARD9* mutation site was also identical with that of P1. However, unlike sclerotic bodies observed in the P1 tissue, the morphology of fungus in P2 tissue had short, septate, and dematiaceous hyphae, which supported the diagnosis of phaeohyphomycosis.

### *CARD9* mutation compromised cytokine production and adaptive immune responses in PBMCs from both patients

**Figure 2:**
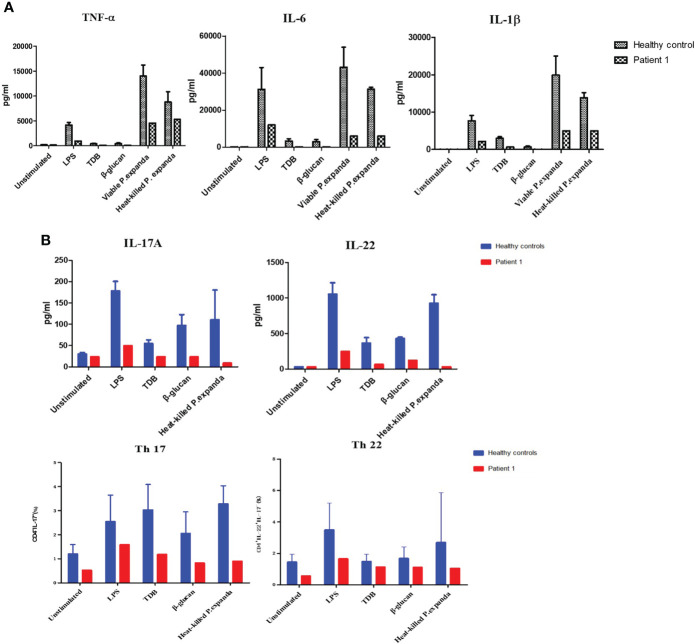
Functional studies of patient 1. **(A)** Detection of natural immune responses in peripheral blood mononuclear cells (PBMCs) separated from the patient and healthy controls (n = 3). Pro‐inflammatory cytokines production of TNF‐α, IL‐6, and IL‐1β in response to 24‐h stimulations with LPS, TDB, β‐glucan, and *P. expanda* conidia (HK or viable resting) was detected in the supernatants by commercial ELISA kits **(B)** Detection of acquired immune responses in PBMCs separated from the patient and healthy controls. Cytokine production of IL‐17A, IL-22 in response to LPS, TDB, β‐glucan, and *P. expanda* conidia (HK) stimulations were measured in the cell culture supernatants after 6 days using commercial ELISA kits (n = 3). The proportions of Th17 (IL‐17+CD4+) Th22 (IL‐22+IL‐17‐CD4+) cells in the total number of CD4+ T cells were counted by flow cytometry after 6‐day stimulation (n = 6). LPS, lipopolysaccharide; TDB, Trehalose dibehenate; P1, patient1; Th, T helper type.

Primary peripheral immune cells were isolated from the two patients to determine the functional effects of CARD9 deficiency. We first used PBMCs from the P1 to assess the release of inflammatory cytokines (TNF-α, IL-6, and IL-1β) in response to 24 h stimulations with LPS, TDB, β‐glucan, and fungal particles, including *P. expanda* conidia (HK or viable yeast). Compared with cells isolated from the healthy control group, the P1 showed comparable defects in IL-6, IL-1β, and TNF-α expression in response to stimulations of LPS, TDB, β‐glucan, and HK or viable *P. expanda* conidia (**Figure 2A**). We then monitored Th cell responses in the patient and the control PBMCs stimulated with HK fungi for 6 days and found a marked attenuation in IL-22 and IL-17 expression in the patient’s cells. Consistently, the patient’s samples showed a significant reduction in Th22 (IL-22^+^IL-17^-^CD4^+^) and Th17 (IL-17^+^CD4^+^) cells (**Figure 2B**). Similar data were obtained in P2 in our previous report, suggesting comparable deficient ex vivo immune responses in these two patients (6).

### Comparative transcriptome sequencing revealed different local immune responses in these patients

**Figure 3:**
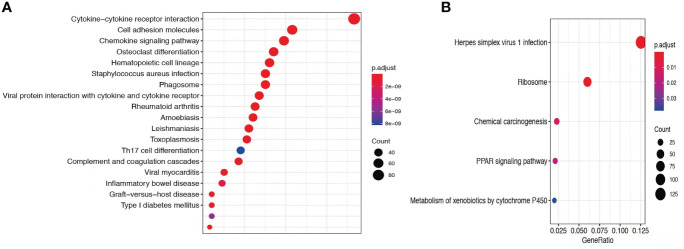
Comparative transcriptome sequencing of patient 1. **(A)** Up-regulated DEGs in P1 were enriched antifungal immune pathways. **(B)** Dot diagram showed the enriched pathways in P1. Color of nodes corresponding to significant levels. Size of nodes represented the number of genes in certain pathway.

**Figure 4:**
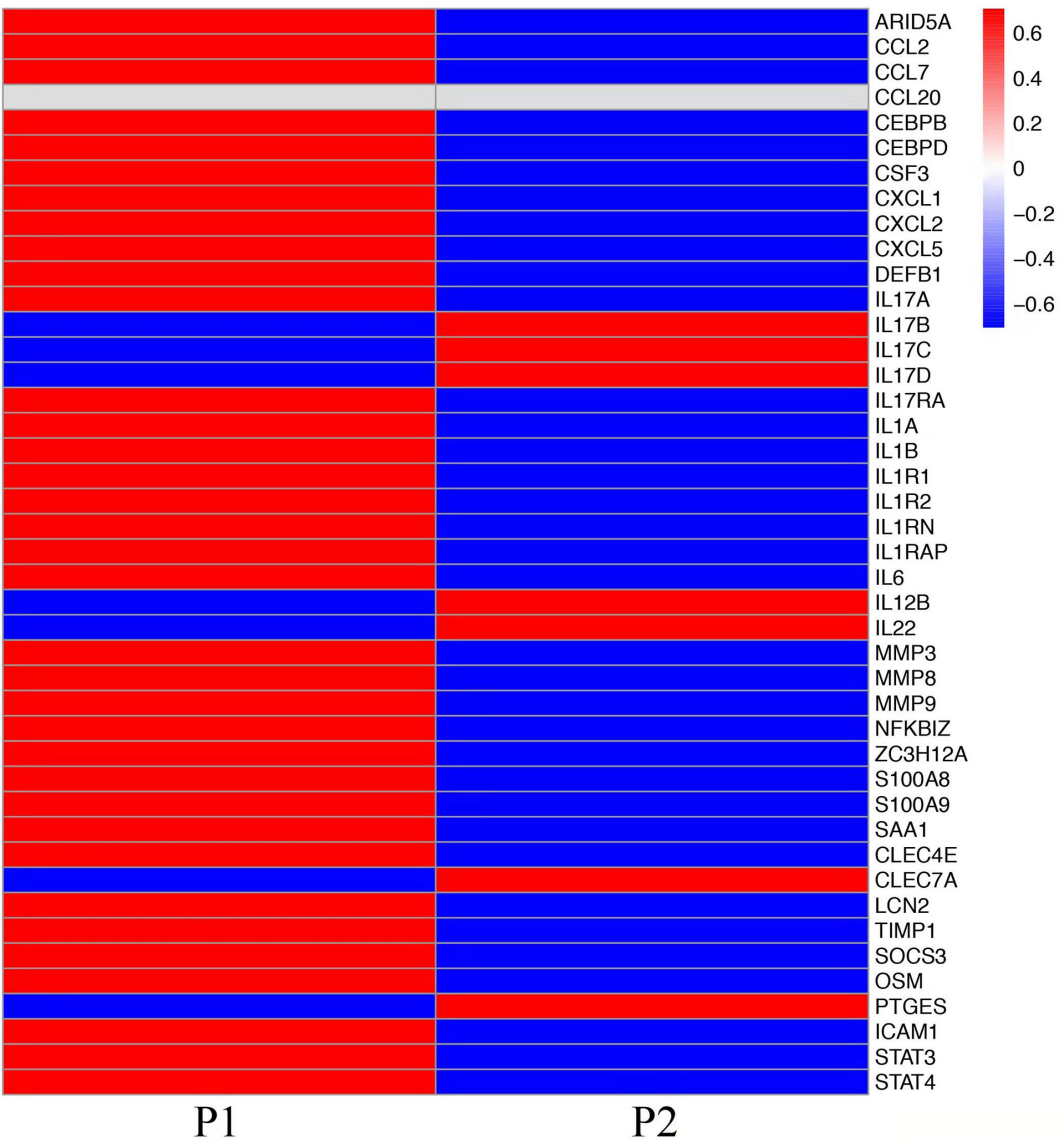
Comparative transcriptome sequencing in these two patients. Heatmap showed the transcript viabilities of antifungal immunity-related genes. Red represented high viability and blue represented low activity.

To explore the mechanisms of heterogeneous fungal morphotypes in the two patients, we performed RNA-seq analysis using the biopsy tissue from the lesions. A total of 4,935 DEGs (P1 versus P2) including 1,972 up-regulated and 2,963 down-regulated DEGs were screened out. Up-regulated DEGs were enriched in several antifungal-related pathways, including cytokine-cytokine receptor interaction, chemokine signaling pathway, and IL-17 signaling pathway (**Figure 3**), indicating that *P. expanda* induces higher excessive immune response, thus P1 had a relatively higher inflammatory response than P2. However, down-regulated DEGs only were enriched in the Herpes simplex virus 1 related pathway, Ribosome and Chemical carcinogenesis, and a few irrelevant pathways (**Figure 3**), suggesting that *P. americana* caused a relatively weak inflammatory response. Accordingly, pattern recognition related genes (CLEC4E), proinflammatory cytokines related genes (IL-1β, IL-6), chemokine related genes (CXCL1, CXCL2), antimicrobial peptides related genes (S100A8, MMP, DEFB1), and adaptive immunity-related genes (IL-17A, IL-17RA), all showed higher expression in P1 versus P2 (**Figure 4**). Given the heterogeneous pathway activities between the two patients, we speculate that local immune cell proportions are also different. Indeed, we found that P1 showed higher CD68, CD11c, MPO, CD4 expression by immunohistochemistry, indicating more macrophages, dendritic cells, neutrophils and CD4^+^ T cells infiltrated to the lesion, respectively (**Supplementary Figure 1**). Different infiltration of the immune cells may in return explain the stronger responses induced by *P. expanda* than *P. americana*. Together, these results indicate that these two pathogens induce different immune responses during infections in patients with the same gene mutations.

### *Phialophora expanda* causing chromoblastomycosis in *Card9* KO mice and *in vitro* validation

**Figure 5:**
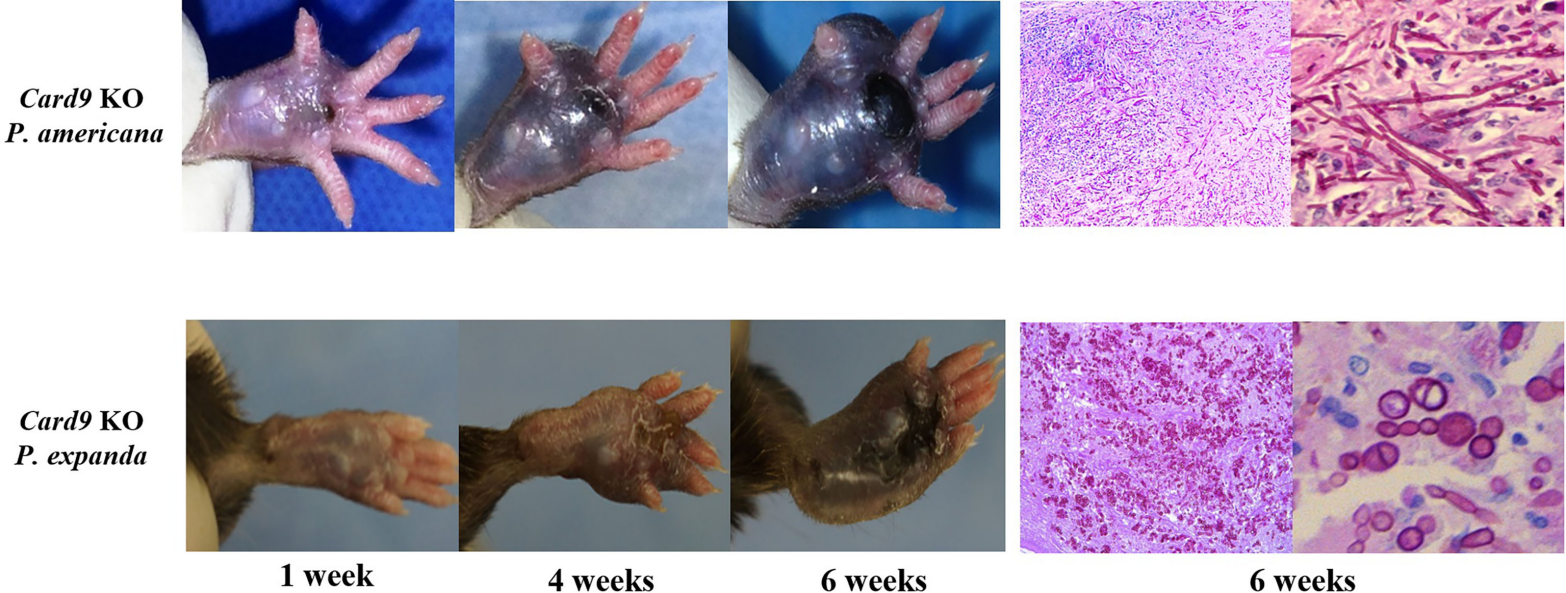
Murine model of subcutaneous dematiaceous fungal infections. *Card9* KO mice were inoculated subcutaneously with 1×10^8^ CFU of live *P. expanda* or *P. americana* spores. Representative images of infected footpads (1/4/6 weeks post-inoculation). Histopathology of PAS-stained footpad from infected *Card9* KO mice at 6 weeks post-infection (original magnification ×200/400).

To study fungal specificity in these two species, we carried out *in vivo* and *in vitro* studies to test the morphotypes of them. We first used *Card9* KO mice and inoculated fungal spores (*P. expanda* and *P. americana*) into the footpad, as previously described (3, 6). The results revealed that both *P. expanda* and *P. americana* were able to cause chronic infections in *Card9* KO mice, whereas WT mice recovered from the inoculation in 4 weeks (**Figure 5**). Similar lesions of swelling, abscess, ulceration, and crust were noted in these two isolates infected mice at 6 weeks post-infection. However, unlike plenty of pigmented fungal hyphae in *P. americana* infected mice, *P. expanda* infected mice showed lots of sclerotic bodies of fungal elements in tissue (**Figure 5**). These results indicated that *P. americana* is prone to causing phaeohyphomycosis, whereas *P. expanda* causing chromoblastomycosis in *Card9* KO mice.

**Figure 6:**
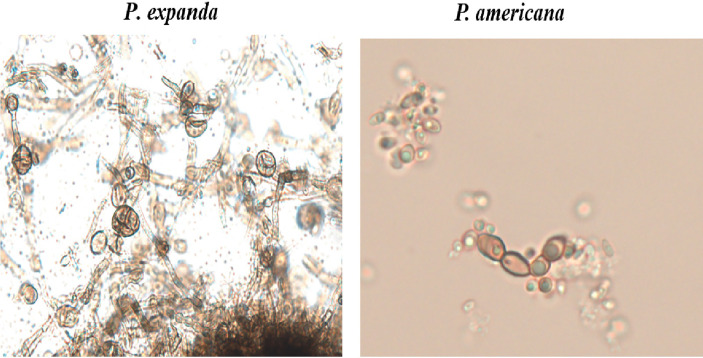
*In vitro* induction of muriform cells. Induction experiments *in vitro* to induce muriform cells (original magnification ×400). After 50 days *P. expanda* was easier to form muriform cells, but *P. americana* was beaded hyphae.

For further verification, we designed induction experiments *in vitro* to compare the ability to form muriform cells in these two isolates. After 50 days of culture, we successfully induced muriform cells in *P. expanda*, whereas *P. americana* showed beaded hyphae. Therefore, we successively confirmed that *P. expanda* is prone to forming a sclerotic body both *in vivo* and *in vitro*, which is consistent with our clinical findings (**Figure 6**).

## Discussion

Dematiaceous fungi have been reported to cause subcutaneous and invasive infections, including chromoblastomycosis, phaeohyphomycosis, and mycetoma (6). Phaeohyphomycosis is increasingly being seen in both immunocompetent and immunocompromised hosts. Invasive phaeohyphomycosis is associated with a poor prognosis, with a case fatality close to 80% (7). In the past decade, mutations in *CARD9* have been found to be the cause of various fungal infections. CARD9 is a critical adaptor of pattern recognition receptors, which includes dectin-1, dectin-2, dectin-3, and mincle. It might also be involved in the response to intracellular danger sensors, such as Nod2, and it has been considered as a bridge that links innate and adaptive immunity in the host defense against fungi (6, 8, 9). As a key adaptor molecule in the downstream signaling of several C-type lectin receptors, CARD9 deficiency may lead to different fungal infections, including mucocutaneous or invasive candidiasis, deep dermatophytosis, phaeohyphomycosis, extrapulmonary aspergillosis, mucormycosis and trichosporosis (10–15). In our previous study, we reported 9 patients with phaeohyphomycosis, and autosomal recessive *CARD9* mutations were identified in all of them (3, 6, 15). As for chromoblastomycosis, it is a chronic dermatosis that mainly affects the lower limbs and usually occurs following transcutaneous wounding caused by plants. The prognosis of chromoblastomycosis is relatively favorable since the systematic spread is rare. Still, its pathogenesis is unclear, and there are no reports of genetic susceptibility (16, 17).

To our knowledge, our present patient (P1) is the first case that links chromoblastomycosis with *CARD9* mutation. Through the PBMCs experiments *in vitro*, we detected that the secretion of inflammatory cytokines and the differentiation of Th cells in this patient were significantly impaired, which were similar to the other phaeohyphomycosis patients with *CARD9* mutations reported previously (3, 6, 15). Although we could not rule out the involvement of other genes or gender factors, it is interesting that these two patients (P1 and P2), with the same *CARD9* mutation site, and similar PBMCs responses, harbored diverse fungal morphology in tissue. Hence, we conjectured that fungal specificity might also contribute to the clinical phenotype in CARD9 deficient patients with dematiaceous fungal infections.

To study the mechanism of heterogeneous clinical manifestation between the two patients, we performed RNA-seq to compare the local immune responses. Based on our results, P1 showed relatively higher and broader local immune responses than P2, both from enriched pathways and from the expression of antifungal immunity-related genes. In line with the above results, more pro-inflammatory immune cells (including macrophages, histocytes, dendritic cells, neutrophils and CD4^+^ T cells) infiltrated in P1. We realized that this is only a relative comparison. Higher immune responses in P1 do not indicate normal and complete antifungal immune responses, which we would not expect to see in CARD9 deficient patients. However, with the same mutation site, and similar *ex vivo* immune responses, *P. expanda* showed higher immune responses locally, and the presence of sclerotic bodies. Thus, we inferred that the different local immune responses and different morphotypes might be related with species specificity.

Based on the above findings, we speculate that *P. expanda* is more likely to form sclerotic bodies and has a stronger immunogenicity than *P. americana*, which also exacerbates this environmental selection and in return turns the conidia into sclerotic bodies. To verify our hypothesis, animal experiments *in vivo* and induction experiments *in vitro* were carried out. Remarkably, although they were from the same complex, these two isolates also showed different morphology in *Card9* KO mice, which is very similar to those seen in patients. Besides, we also successfully induced sclerotic bodies of *P. expanda in vitro*, whereas failed in the case of *P. americana*. Considering the inconsistent size of sclerotic cells and inconvenient counting, we did not use it as a stimulation. In summary, our preliminary data showed that *P. expanda* is prone to forming sclerotic bodies, which might explain the different morphotypes and different local responses in our patients.

## Conclusion

We report the case of a patient with chromoblastomycosis harboring a *CARD9* mutation. This is, to our knowledge, the first report that links chromoblastomycosis to *CARD9* mutation, which challenges our previous perspective that chromoblastomycosis patients are mostly immunocompetent. We showed that, besides host immune responses, fungal specificity is also closely involved in shifting the clinical phenotype in CARD9 deficient patients with dematiaceous fungal infections. We hope that this preliminary research will be beneficial to clinicians and will call for additional efforts to systematically study these uncommon, frequently refractory infections and their underlying genetic background.

## Data availability statement

The datasets presented in this study can be found in online repositories. The names of the repository/repositories and accession number(s) can be found below: https://ngdc.cncb.ac.cn/, HRA002793.

## Ethics statement

The studies involving human participants were reviewed and approved by The Clinical Research Ethics Committee of the Peking University First Hospital. The patients/participants provided their written informed consent to participate in this study. The animal study was reviewed and approved by The Animal Research Ethics Committee of the Peking University First Hospital.

## Author contributions

CH, WD and YZ conducted the research, and analyzed and interpreted the data. KZ and YM participated in animal experiments. ZW and YS contributed with the identification of the fungus. XW and RL are the principal investigators who conceived this study. CH drafted the manuscript and XW and RL critically revised the manuscript. All authors contributed to the article and approved the submitted version.

## Funding

This work was supported by the National Natural Science Foundation of China (NSFC No. 81872539) and Jiangsu Innovative & Enterpreneurial Talent Programme (JSSCBS20211463).

## Acknowledgments

We are grateful to the two patients, their family members, and the healthy donors for their participation in this study.

## Conflict of interest

The authors declare that the research was conducted in the absence of any commercial or financial relationships that could be construed as a potential conflict of interest.

## Publisher’s note

All claims expressed in this article are solely those of the authors and do not necessarily represent those of their affiliated organizations, or those of the publisher, the editors and the reviewers. Any product that may be evaluated in this article, or claim that may be made by its manufacturer, is not guaranteed or endorsed by the publisher.
