# Supplementary material for: CARD9 deficiency predisposing chromoblastomycosis: A case report and comparative transcriptome study
Source: Front Immunol. 2022 Sep 9;13:984093. doi: 10.3389/fimmu.2022.984093 (PMC9500462; doi:10.3389/fimmu.2022.984093)
Supplement: Supplementary file 1 [file DataSheet_1.docx]

Supplementary Material


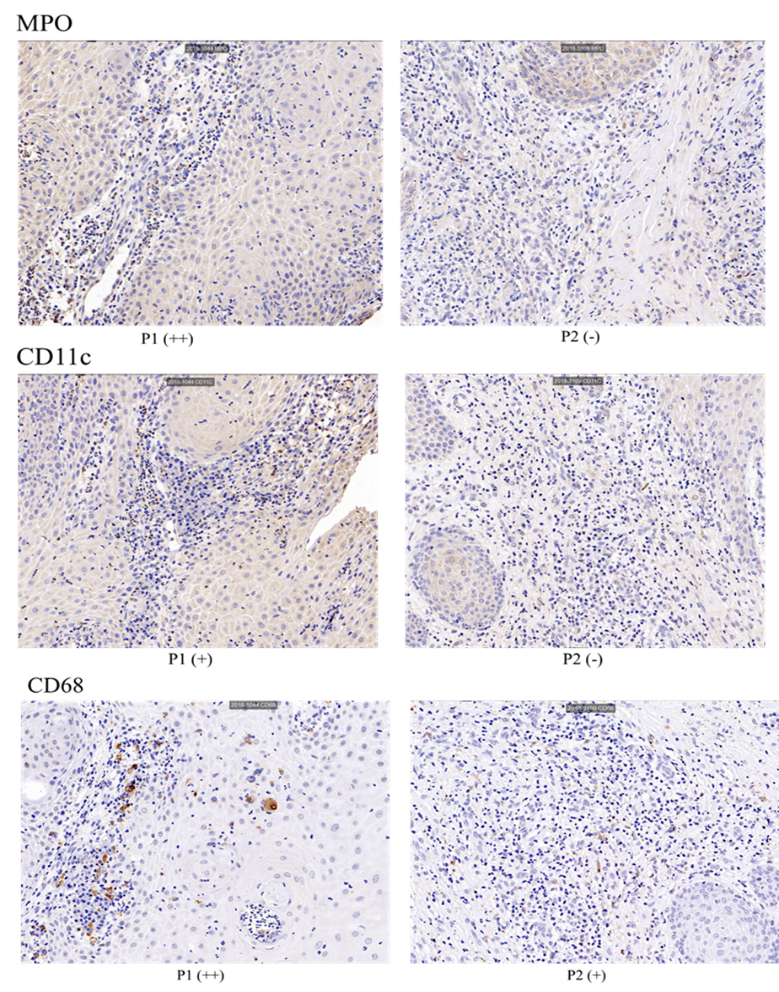


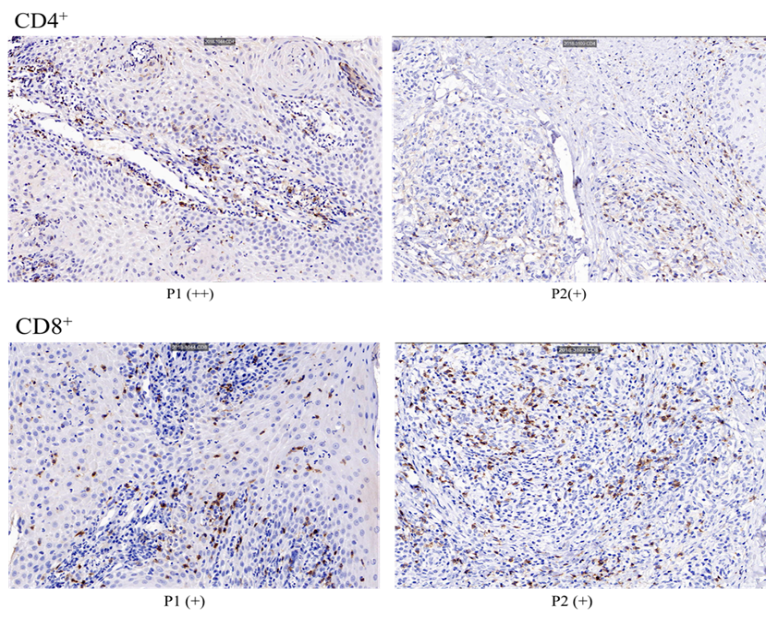


**Supplementary Figure 1.** Immunohistochemical analysis of the two patients skin lesion with different marker to demonstrate the local immune cells infiltration (original magnification ×200).
